# Supraglottic jet oxygenation and ventilation improves oxygenation during endoscopic retrograde cholangiopancreatography: a randomized controlled clinical trial

**DOI:** 10.1186/s12871-024-02406-y

**Published:** 2024-01-12

**Authors:** Dan Su, Wei Zhang, Jingze Li, Xi Tan, Huafeng Wei, Yinglin Wang, Zhonghua Ji

**Affiliations:** 1 Department of Anesthesia, Shanghai East Hospital, Tongji University School of Medicine, Shanghai, 200120 China; 2 Endoscopy Center, Department of Gastroenterology, Shanghai East Hospital, Tongji University School of Medicine, Shanghai, 200120 China; 3 Department of Anesthesiology and Critical Care Medicine, University of Pennsylvania, Philadelphia, PA 19104 USA

**Keywords:** Endoscopic retrograde cholangiopancreatography, Hypoxia, Deep sedation, Ventilation, Semiprone position

## Abstract

**Purpose:**

Hypoxia is one of the most frequent adverse events under deep sedation in the semiprone position. We hypothesized that supraglottic jet oxygenation and ventilation (SJOV) via Wei nasal jet tube (WNJ) can reduce the incidence of hypoxia in patients under deep sedation during endoscopic retrograde cholangiopancreatography (ERCP).

**Methods:**

A total of 171 patients were divided into three groups: N group, supplementary oxygen via a nasopharyngeal airway (4–6 L/min); W group, supplementary oxygen via WNJ (4–6 L/min); WS group, SJOV via WNJ. The primary outcome was the incidence of adverse events, including sedation-related adverse events [SRAEs, hypoxemia (SpO_2_ = 75–89% lasted less than 60 s); severe hypoxemia (SpO_2_ < 75% at any time or SpO_2_ < 90% lasted more than 60 s] and subclinical respiratory depression (SpO_2_ = 90–95%). Other intraoperative and post-operative adverse events were also recorded as secondary outcomes.

**Results:**

Compared with the N group, the incidence of hypoxemia and subclinical respiratory depression in the WS group was significantly lower (21% vs. 4%, *P* = 0.005; 27% vs. 6%, *P* = 0.002). Compared with Group W, the incidence of hypoxemia and subclinical respiratory depression in Group WS was also significantly less frequent (20% vs. 4%, *P* = 0.009; 21% vs. 6%, *P* = 0.014). No severe hypoxia occurred in the group WS, while four and one instances were observed in the group N and group W respectively. There were no significant differences in other adverse events among the three groups.

**Conclusion:**

SJOV can effectively improve oxygenation during ERCP in deeply sedated semiprone patients.

**Supplementary Information:**

The online version contains supplementary material available at 10.1186/s12871-024-02406-y.

## Introduction

Endoscopic retrograde cholangiopancreatography (ERCP) is a technique for evaluating the bile duct, pancreatic duct, and ampulla. With the development of endoscopic sphincterotomy in recent years, ERCP has evolved from a purely diagnostic imaging method into a way to perform both diagnostic and therapeutic procedures [1]. Compared with other endoscopic procedures, ERCP is more invasive; thus, comparatively deep sedation to prevent uncontrolled movements and coughing is often required to meet the procedure requirements [2].

Hypoxia is the most common cardiopulmonary complication during ERCP, with a reported rate of 16.2 to 39.2% [3]. A left-semiprone position, which is often used in ERCP procedures, will lead to hypoventilation for decreased chest wall compliance, reduced functional residual capacity (FRC) and suppressed breathing [4]. Besides, propofol and opioids are likely to result in respiratory depression and airway obstruction, leading to hypoxia [5]. Therefore, it is critically important to prevent hypoxia during sedation for patients’ safety and procedural success in ERCP [6].

The key to preventing hypoxia is to ensure the sufficient oxygenation and ventilation of patients during these procedures. The commonly used approaches to treat hypoxia with a non-instrumented airway are increasing the oxygen flow and lifting the jaw, applying with both hands, displacing the jaw upwards and anteriorly, which allowed the upper airway to remain open [7]. The placement of a nasopharyngeal airway may be a good approach to prevent hypoxia, by keeping the airway open, while it is difficult to detect hypoventilation promptly when patients are under suppressed breathing [3, 4]. Tracheal intubation devices could provide mechanical ventilation by connecting to ventilators, but may take up the space of duodenal rectoscope, which would interfere with duodenoscopy procedures [8]. Previous research has confirmed that supraglottic jet oxygenation and ventilation (SJOV) through a new Wei nasal jet tube (WNJ) enhances oxygenation during upper gastrointestinal endoscopy in sedated patients [9–12]. However, as ERCP is more invasive, requiring deeper sedation and longer duration of procedure, which means higher incidence of hypoxia, it is not very clear the efficacy of SJOV through WNJ to prevent hypoxia in deeper sedated patients during the longer procedure.

This study was a prospective, single-blinded and randomized controlled clinical study. We hypothesized that SJOV via WNJ could prevent hypoxia in patients under deep sedation during ERCP.

## Methods

### Ethics, clinical trial, consent and permissions

This clinical study was approved by the Institutional Review Board and Ethics Committee of Affiliated East Hospital of Tongji University (2021, No.097), Shanghai, China (Chairperson Prof Zengguang Xu) on 5 November 2021, and was registered at chictr.org.cn (ChiCTR2100053532, https://www.chictr.org.cn/showprojEN.html?proj=139528, Principal investigator: Zhonghua Ji, Date of registration: 2021.11.24). The clinical trial was registered prior to patient enrollment. The written informed consent was obtained from all subjects participating in the trial. This study followed the Consolidated Standards of Reporting Trials (CONSORT) guidelines and was conducted in accordance with the Helsinki Declaration-2013.

### Patient inclusion and exclusion

Inclusion criteria were as follows: (1) Ages from 18 to 65 years; (2) American Society of Anesthesiologists (ASA) classification from I to III; (3) The general state was stable, and the respiratory function reserve was good; (4) Procedure time < 2 hours. Exclusion criteria were as follows: (1) Coagulopathy or epistaxis; (2) Body mass index (BMI) over 28 kg/m^2^; (3) Concomitant severe heart diseases (heart failure, angina pectoris, myocardial infarction, arrhythmia, etc.); (4) Concomitant severe lung diseases (asthma, chronic obstructive pulmonary disease, pulmonary embolism, pulmonary oedema or lung cancer, etc.); (5) Pregnancy; (6) Increased intracranial pressure; (7) Infection of the nasal cavity, oropharynx, or other contraindication to insert the nasopharyngeal airway and WNJ, such as nasal surgery, etc.; (8) Allergy to propofol, egg, soy or albumin. Withdrawal criteria as follows: (1) Procedure time more than 2 hours; (2) Undergoing angiography instead of treatment; (3) Failure to insert the nasopharyngeal airway and WNJ; (4) Failure to follow up; (5) Failure to finish the trial for other reasons.

### Randomization, group allocation and blinding

Patients were randomized into three groups based on the approaches of oxygen supplies: the supplementary oxygen via a nasopharyngeal airway group at an oxygen flow of 4–6 L/min (N group), the supplementary oxygen at 4–6 L/min via the WNJ (WNJ; Well Lead Medical Company Ltd., Guangzhou, China) (W group), or the SJOV via the WNJ (SJOV working parameters: the driving pressure is 15 psi, the breathing rate is 20 breath per minute, the inspiratory to expiratory ratio is 1:2, the gas supply was maintained for 5 min after inserting the WNJ successfully, and the concentration of oxygen is 100%. Apart from the SJOV, the supplementary oxygen was at 4–6 L/min via WNJ) (WS group). Internet-based randomization software (http://www.randomization.com) was used for randomization. Patients were allocated blindly after randomization. Other individuals who participated in the research were not blinded to the group assignments. The data were collected by a resident.

### Sample size calculation

PASS software (version 15.0, NCSS, LLC, Kaysville, UT, United States) was used to determine the sample size. The contingency table (chi-square test) was performed for multiple comparison of proportion. When α was 0.05, the test power was 80%, the effect size was 0.25, and the degree of freedom was 2, we calculated that 155 patients were needed. The attrition rate was set at 10%, requiring a total of 171 patients (57 of each group). The effect size was calculated based on the assumption derived from our pretest study that the incidence of hypoxemia (SpO_2_ = 75–89% lasted less than 60 s) during ERCP under deep sedation with SJOV via WNJ was 5%. The incidence of hypoxemia during ERCP for patients who were oxygenated with a nasopharyngeal airway was 25% in our preliminary experiments.

### Procedure and sedation strategy

Sedation was performed by a team that involved attending anesthesiologists and nurse anesthetists. All patients were fasted for 6 h for food and 2 h for clear liquid prior to the procedure. After entering the endoscopy suite, venous access was established, and the vital signs of the patients were monitored, including electrocardiograph (ECG), peripheral oxygen saturation (SpO_2_), heart rate (HR), noninvasive systolic arterial pressure (SAP), diastolic arterial pressure (DAP), mean arterial pressure (MAP), respiratory rate (RR), bispectral index (BIS), and end-tidal carbon dioxide partial pressure (P_ET_CO_2_). Patients were positioned in the semiprone position with the right side elevated with a chest pillow.

**Fig. 1:**
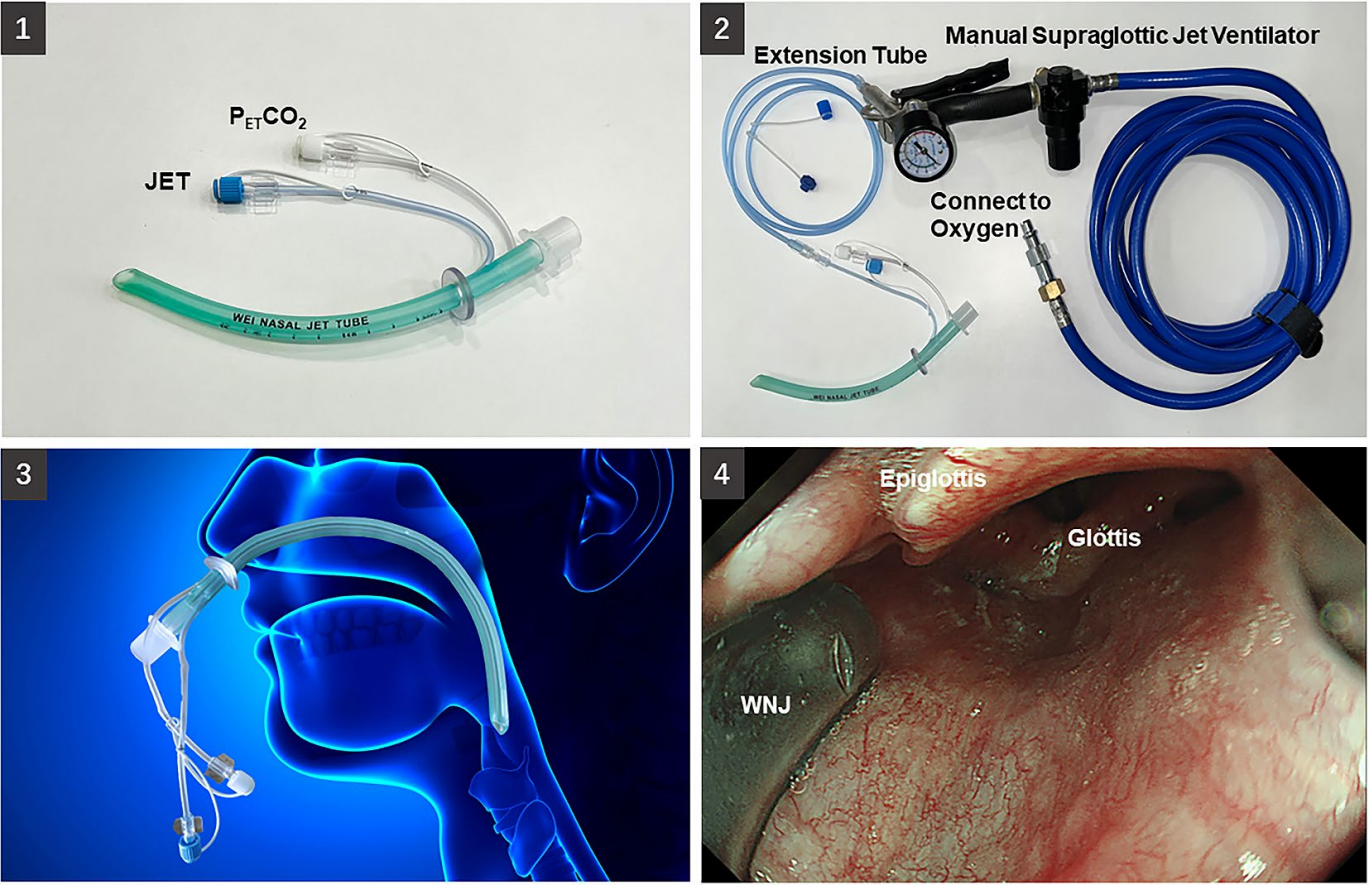
(**1**) Wei nasal jet tube (WNJ) with a manual jet ventilator and end-tidal CO_2_ pressure (P_ET_CO_2_) monitoring. (**2**) The supraglottic jet oxygenation and ventilation (SJOV) via a WNJ connected to a manual jet ventilator. (**3**) The position of WNJ in patients. (**4**) The best position of the WNJ tip is between the epiglottis and uvula

Before placing the nasopharyngeal airway or WNJ, a cotton swab with saline was used to clear the nasal cavity, and ephedrine (0.6%, 2 ml) and lidocaine (2%, 5 ml) were then sprayed to numb the nasal vestibule and passage to reduce nose bleeding [13]. The tip of the nasopharyngeal airway or WNJ [Fig. 1(1)] was lubricated with 1 ml paraffin oil. The depth of placement was roughly equivalent to the distance from the alar of the nose to the earlobe on the same side [9]. Then, we started placing the nasopharyngeal airway or WNJ at the appropriate depth as measured in advance, its position was affirmed again by the duodenal rectoscope [Fig. 1(3, 4)], and appropriate adjustments were performed if necessary. When there were difficulties in inserting the nasopharyngeal airway or WNJ via the selected naris, the other naris was available for further attempts. After three failures, the placement of nasopharyngeal airway or WNJ was considered to be failed.

In group N, nasal cannula was used to supply oxygen through nasopharyngeal airway. In group W and WS, the connector of the WNJ was connected to the threaded pipe joint of the anesthesia machine (Aespire 7100, Datex-Ohmeda, Inc., Madison, WI, USA) for oxygen supply. The CO_2_ catheter of the WNJ was connected to the portable end-tidal carbon dioxide partial pressure monitor (KMI605D, Kingst, Inc., Beijing, China), and the jet catheter of the WNJ in the WS group was connected to a manual jet ventilator (Anesthesia Associate, Inc., San Marcos, CA, USA) [Fig. 1(2)] to conduct supraglottic jet oxygenation and ventilation (SJOV).

Sedation was conducted by intravenous administration of propofol, remifentanil and dexmedetomidine. Dexmedetomidine was infused with a preloading dose of 1 µg/kg for 10 min, and the target blood concentration of remifentanil was set at 1 ng/ml [14]. Propofol infusion was given with a target-controlled infusion (TCI) system composed of an ALARIS ASENA TIVA pump produced by ALARIS and a propofol prefilled syringe produced by Astrazeneca Pharmaceutical Co., Ltd. The initial target blood concentration of propofol was set at 3.0 µg/ml, and adjustments of 1.5–1.7 µg/ml were made to achieve the expected sedation level by monitoring the BIS value from 45 to 60 [15]. The Marsh parameter model [16] was referenced, and the ideal concentrations of propofol and remifentanil were set at 10 mg/ml and 20 µg/ml, respectively. Intravenous premedication with hyoscine 0.3 mg/kg was given to patients in all 3 groups to relieve spasms of the duodenum.

All procedures were provided by an experienced endoscopist (Jingze Li, who had completed more than 200 ERCPs independently) with the assistance of 1 to 3 endoscopic nurses. The endoscopist started the procedure after BIS reached the target value with a standard duodenoscope (TJF 240 or 260 V; Olympus Optical Co., Ltd., Tokyo, Japan). Under the direct vision of the duodenoscope, when the duodenoscope reached near the glottis, the WNJ was adjusted in and out slightly along the nasal cavity to ensure that the tip of the WNJ was directly opposite the glottis.

When SpO_2_ < 90% occurred in Groups N and W, remedial measures were executed sequentially, including (i) adjusting the position of the nasopharyngeal airway or WNJ, (ii) opening the airway with the jawlift manoeuvre, (iii) mask pressurized ventilation, and (iv) removing the choledochoscope and tracheal intubation. When SpO_2_ < 90% occurred in Group WS, the position of the WNJ was adjusted at first, and then application of SJOV via the WNJ was performed again for another 5 min (increasing the rate of jet ventilation to 30 breath per minute and the driving pressure to 20 psi if necessary) until SpO_2_ > 95%. If it did not work, we followed measures (ii-iv) above.

### Outcome measurement

We recorded the clinical indicators of the three groups, including sedation time (time from induction with propofol to opening eyes in response to sound), procedure time (time from endoscope insertion to withdrawal), recovery time [time from drug withdrawal until the patient’s Aldrete score = 8 (This assigns a score of 0, 1 or 2 to activity, respiration, circulation, consciousness and oxygen saturation, giving a maximal score of 10.) and the Aldrete score was assessed at one, five and ten minutes after the procedure by a nurse anesthetist in the PACU)] [17], total propofol dosage, total remifentanil dosage, and total dexmedetomidine dosage. Primary intraoperative adverse events were divided into two categories: sedation-related adverse events (SRAEs) and subclinical respiratory depression (SpO_2_ = 90–95%) [9]. SRAEs included hypoxemia (SpO_2_ = 75–89% lasted less than 60 s), severe hypoxemia (SpO_2_ < 75% at any time or SpO_2_ < 90% lasted more than 60 s) and implementation of the above emergency measures [18, 19].

Other intraoperative adverse events included cough, laryngospasm, muscle twitch, tachycardia (heart rate more than 100 bpm), bradycardia (heart rate less than 60 bpm), hypertension (blood pressure increased to more than 20% of baseline), hypotension (blood pressure decreased to more than 20% of baseline) [15], and body movement. Postoperative adverse events involved nose bleeding, nausea or vomiting, recovery delay (delayed recovery time for more than 30 min), dysphoria, xerostomia, pharyngalgia, barotrauma, and airway injury were recorded at 5, 30 min and 24 h after the procedure.

### Statistical analysis

Measurement data are presented as the mean (SD), and count data are presented as the number and percentage. One-way ANOVA or the Pearson χ^2^ test was used to compare differences in the general data of patients according to the type of data. The differences in sedation time, procedure time, recovery time, and dosage of anaesthetics were tested by the Kruskal‒Wallis H test between different groups. Dunn’s z test was used to compare the differences among the three groups when *P* < 0.05. The χ^2^ test and Fisher’s exact test were used to analyse the rates of SRAEs and other adverse events. A χ^2^ test and Fisher’s exact test were used to analyse the adverse event incidence rate. As three χ^2^ test were performed, the *P*-value was adjusted to 0.05/3 *≈* 0.017 by Bonferroni adjustment.

## Results

**Fig. 2:**
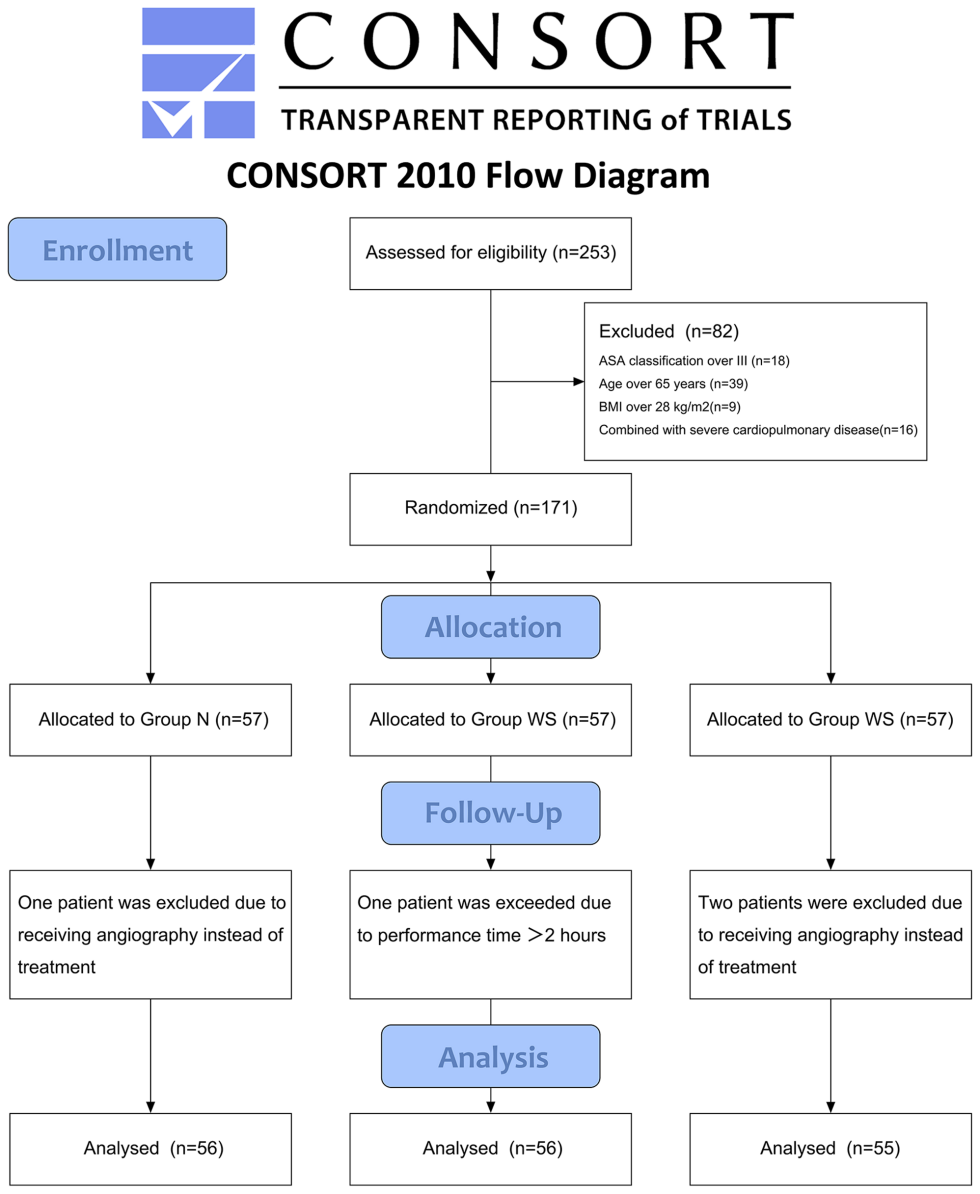
Consolidated standards of reporting trial flow diagram. BMI, body mass index; ASA, American Society of Anesthesiologists; Group N, the supplementary oxygen via nasopharyngeal airway group; Group W, the supplementary oxygen via WNJ group; Group WS, SJOV via WNJ group

A total of 253 patients were assessed for eligibility, but 18 patients were classified as ASA more than III, 39 patients were aged over 65 years, 9 patients had a BMI over 28 kg/m^2^, and 16 patients had severe cardiopulmonary diseases. In total, 171 patients were enrolled and equally randomized to three groups. There were 1 and 2 patients excluded due to receiving only diagnostic cholangiogram but not treatment in Group N and Group WS, respectively, and 1 patient in Group W was excluded due to the procedure lasting more than two hours. In total, 167 patients were included in the final statistics. All of the patients tolerated the procedure well. The insertion of the nasopharyngeal airway and WNJ were both successful. No severe adverse events, such as aspiration, laryngospasm, barotrauma and death, occurred (Fig. 2).

### Clinical characteristics of the study population

General patient information is shown in Table 1. Age, sex, BMI, history of hypertension, diabetes and snoring, Mallampati class, thyromental distance, obstructive sleep apnoea hypoventilation syndrome (OSAHS), ASA, and baseline SpO_2_ were compared. Data about the procedure time and the dosage of anesthetics are given in Supplementary Table 1. Compared with Group N, the use of SJOV via WNJ (Group WS) decreased the procedure time from 34.13(4.03) mins to 32.27 (3.73) mins (*P* = 0.013); there was no significant difference between Group W and Group WS. However, the sedation time and recovery time among the three groups showed no significant differences. Furthermore, there was no significant difference in the total doses of propofol, dexmedetomidine and remifentanil among the three groups.

**Table 1:** General characteristics of patients

Characteristic	Group N (n = 56)	Group W (n = 56)		Group WS (n = 55)
Age[yr;mean(range)]	56(39–65)	56(43–65)		56(40–65)
Sex(male)(female)	(22)(34)	(27)(29)		(25)(30)
BMI[kg/m^2^;mean(SD)]	23(2.97)	22(2.30)		23(2.27)
History of hypertension [n(%)]	23(41)	21(38)		24(44)
History of diabetes[n(%)]	22(39)	15(27)		13(24)
Mallampati class (I)(I)(III)(IV)	(28)(21)(6)(1)		(22)(18)(15)(1)	(22)(16)(15)(2)
Thyromental distance [cm;mean(SD)]	7(0.29)	7(0.27)		7(0.37)
History of snoring [n(%)]	18(32)	14(25)		18(33)
OSAHS[n(%)]	1(2)	0(0)		0(0)
ASA(I)(II)(III)	(7)(33)(16)	(5)(35)(16)		(2)(33)(20)
SpO_2_ before sedation [%;mean(SD)]	98(1.28)	98(1.53)		98(1.36)

BMI, body mass index; OSAHS, obstructive sleep apnea–hypopnea syndrome; ASA, American Society of Anesthesiologists; SpO_2_, peripheral oxygen saturation

### Primary outcome

SJOV via WNJ significantly decreased the incidence of SRAEs during ERCP under deep sedation (Table 2). Compared with Group N, the application of SJOV via WNJ significantly decreased the total percentage of SRAEs from 29 to 4% (*P* = 0.000), decreased the incidence of subclinical respiratory depression from 27 to 6% (*P* = 0.002), and decreased the incidence of hypoxemia from 21 to 4% (*P* = 0.005). Correspondingly, compared with Group N, the incidence of jawlift in Group WS significantly decreased (*P* = 0.003). None of the patients in Group WS required mask ventilation. Compared with Group W, Group WS had a decreased incidence of SRAEs from 21 to 4% (*P* = 0.005) and a decreased incidence of hypoxemia, subclinical respiratory depression, and jawlift from 20 to 4% (*P* = 0.009), from 21 to 6% (*P* = 0.014), and from 20 to 4% (*P* = 0.009), respectively. Furthermore, there were no significant differences in episodes of severe hypoxemia and mask ventilation between Group W and Group WS (*P* = 1.000, *P* = 0.057). There were two patients in Group N who needed tracheal intubation, while the rest of the patients in the three groups finished ERCP without tracheal intubation.

**Table 2:** Sedation-related adverse events (SRAEs) and subclinical respiratory depression

	Group N(I) (n = 56)	Group W(II) (n = 56)	Group WS(III) (n = 55)	*P*-value (I vs. II)	*P*-value (II vs. III)	*P*-value (I vs. III)
Subclinical respiratory depression[n(%)]	15(27)	12(21)	3(6)	0.508	0.014	0.002
**SRAEs[n(%)]**	16(29)	12(21)	2(4)	0.383	0.005	0.000
Hypoxemia	12(21)	11(20)	2(4)	0.815	0.009	0.005
Severe hypoxemia	4(7)	1(2)	0(0)	0.364	1.000	0.118
P_ET_CO_2_<10mmHg[n(%)]	-	13(23)	4(7)	-	0.020	-
Jaw lift[n(%)]	13(23)	11(20)	2(4)	0.645	0.009	0.003
Mask ventilation[n(%)]	5(9)	5(9)	0(0)	1.000	0.057	0.057
Endotracheal intubation[n(%)]	2(4)	0(0)	0(0)	0.495	-	0.495

Subclinical respiratory depression: SpO_2_ = 90–95%, SRAEs: SpO_2_ < 90% and the implementation of emergency measures; P_ET_CO_2_: End-tidal carbon dioxide partial pressure. The χ^2^ test and Fisher’s exact test were used to analyze the incidence rate of SRAEs. The *P*-value was adjusted to 0.05/3 ≈ 0.017 by Bonferroni adjustment

### Secondary outcomes

Compared with Group N, the application of SJOV via WNJ significantly increased the incidence of xerostomia from 2 to 20% (*P* = 0.002) at 5 min after the procedure, but there were no significant differences between two groups at 30 min and 24 h after the procedure (Table 3). There were no significant differences in the incidence of other intraoperative adverse events except SRAEs and subclinical respiratory depression among the three groups. There were no differences in the incidence of other postoperative adverse events, including nausea, vomiting, dysphoria, and pharyngalgia among the three groups. None of the patients suffered from barotrauma or airway injury in any group (Table 4).

**Table 3:** Intraoperative adverse events except SRAEs and subclinical respiratory depression

	Group N(I) (n = 56)	Group W(II) (n = 56)	Group WS(III) (n = 55)	*P*-value (I vs. II)	*P*-value (II vs. III)	*P*-value (I vs. III)
Cough[n(%)]	2(4)	1(2)	1(2)	1.000	1.000	1.000
Laryngospasm[n(%)]	0(0)	0(0)	0(0)	-	-	-
Muscle twitch[n(%)]	7(13)	5(9)	3(6)	0.541	0.716	0.321
Bradycardia[n(%)]	6(11)	6(11)	8(15)	1.000	0.543	0.543
Tachycardia[n(%)]	4(7)	5(9)	6(11)	1.000	0.727	0.527
Hypertension[n(%)]	4(7)	5(9)	2(4)	1.000	0.438	0.679
Hypotension[n(%)]	5(9)	5(9)	4(7)	1.000	1.000	1.000
Body movement [n(%)]	6(11)	5(9)	4(7)	0.751	1.000	0.742

The *P*-value was adjusted to 0.05/3 ≈ 0.017 by Bonferroni adjustment

**Table 4:** Post-operative adverse events

	Group N (I) (n = 56)	Group W (II) (n = 56)	Group WS (III) (n = 55)	*P*-value	*P*-value (I vs. II)	*P*-value (I vs. III)	*P*-value (II vs. III)
**5 min after procedure**							
Nose bleeding (1)(2)(3)	(5)(1)(0)	(13)(2)(0)	(14)(1)(0)	0.547	-	-	-
Nausea or vomiting[n(%)]	10(18)	3(5)	5(9)	0.103	-	-	-
Dysphoria[n(%)]	8(14)	6(11)	5(9)	0.717	-	-	-
Xerostomia[n(%)]	1(2)	3(5)	11(20)	0.001	0.618	0.002	0.018
Pharyngalgia[n(%)]	14(25)	16(29)	18(33)	0.594	-	-	-
Barotrauma[n(%)]	0(0)	0(0)	0(0)	-	-	-	-
Airway injury[n(%)]	0(0)	0(0)	0(0)	-	-	-	-
**30 min after procedure**							
Nose bleeding (1)(2)(3)	(2)(0)(0)	(4)(0)(0)	(7)(0)(0)	0.193	-	-	-
Recovery delay[n(%)]	0(0)	1(2)	0(0)	0.369	-	-	-
Xerostomia[n(%)]	6(11)	8(14)	8(15)	0.765	-	-	-
Pharyngalgia[n(%)]	4(7)	3(5)	8(15)	0.181	-	-	-
**24 h after procedure**							
Nose bleeding[n(%)]	0(0)	0(0)	0(0)	-	-	-	-
Xerostomia[n(%)]	1(2)	0(0)	3(5)	0.151	-	-	-
Pharyngalgia[n(%)]	9(16)	11(20)	9(16)	0.852	-	-	-

Nose bleeding degrees: 1 = mild bleeding, almost no bleeding or slight oozing, 2 = medium bleeding, between 1 and 3, 3 = severe bleeding, endoscopy cannot be fulfilled without suction. The *P*-value was adjusted to 0.05/3 ≈ 0.017 by Bonferroni adjustment

## Discussion

In our study, we aimed to investigate the effectiveness of SJOV via WNJ can reduce the incidence of hypoxia in patients under deep sedation during ERCP. According to our results, SJOV can effectively improve oxygenation during ERCP in deeply sedated semiprone patients. Nasopharyngeal airway can relieve upper airway obstruction to some extent, but for its tip far from glottis, it is too difficult to maintain oxygenation under suppressed breathing. We found that the incidence of hypoxemia was significantly higher in Group N, which is consistent with the results of Han S J [20], while the incidence of hypoxemia was lower in Group W (20%) and Group WS (4%). WNJ can ensure adequate oxygenation under deep sedation, which significantly reduces the incidence of hypoxia compared to the commonly used oxygen supplies via a nasopharyngeal airway [11]. The WNJ is a rapidly inserted device that requires no previous experience or practice to use and it is well tolerated by lightly sedated patients [21]. In our study, each WNJ was placed appropriately in Group W and Group WS. Easy implementation may allow the WNJ with SJOV to be used in emergent airway management as a rescue device [22]. Based on this feature, SJOV via WNJ is considered especially appropriate for the resuscitation of injured people who suffered from acute trauma in war fields [21].

The SJOV system used via WNJ is shown in Fig. 1(2). We confirmed the location of the tip of the WNJ by duodenal rectoscope, concluding that the gap of the epiglottis and uvula was the optimized location for WNJ [Fig. 1(4)]. If the location of WNJ was appropriate, the wave of P_ET_CO_2_ would be regular, and the best position of distal end of WNJ between epiglottis and vulvar usually provide highest P_ET_CO_2_. Oxygenation was better maintained in Group WS than in Group W (Table 2), suggesting that the high-pressure jet pulse promotes ventilation via WNJ. Thus, we inferred that WNJ maintains adequate oxygenation performed primarily by SJOV instead of by recovering spontaneous respiration or relieving an obstructive airway. Furthermore, an animal study confirmed that the SpO_2_ could be maintained over 95% for more than 20 min by SJOV without the need for assisted mask ventilation in an apnoeic pig [23]. The additional utilization of a built-in CO_2_ monitoring catheter on the WNJ may help detect depressed or apnoeic breathing which often occurred after induction of anesthesia. Therefore, SJOV via WNJ lasting for 5 min at the beginning of the procedure has a good effect on relieving hypoxemia caused by hypoventilation. Although SJOV can maintain the desired oxygenation for up to 1 h according to previously reported clinical trials, there is insufficient evidence to support the use of SJOV for a longer duration [24]. In our study, all procedures during ERCP were performed within one hour. We excluded patients whose procedure time exceeded 2 h. The maximum application time and potential airway mucosal inflammation remain to be investigated in the future. The incidences of intraoperative adverse events except SRAEs and subclinical respiratory depression were comparable among all three groups (Table 3). The data on non-invasive arterial pressure and heart rate are listed in Supplementary Table 2, and all patients’ hemodynamic were stable. There was no barotrauma or airway injuries among three groups, and there were no significant differences in the incidence of nose bleeding, nausea or vomiting, dysphoria and pharyngalgia (Table 4). Compared with Group N, the application of SJOV via WNJ significantly increased the incidence of xerostomia from 2 to 20% (*P* = 0.002) at 5 min after the procedure, but there were no significant differences between two groups at 30 min and 24 h after the procedure. The utilization of humidified oxygen could potentially decrease the incidence of xerostomia after using SJOV. As hyoscine was intravenously injected preprocedure, xerostomia may inevitably be attributed to the gland secretion inhibition caused by hyoscine, but it generally disappeared within 30 min postoperatively without any treatment. All of the above complications were tolerable and manageable without difficulties, similar to the results of previous clinical studies [10, 11]. In addition to conveniently using WNJ for SJOV, there are varying techniques reported to perform SJOV, such as the application of a soft-suction catheter [25] and a Cook airway exchange catheter [26]. Compared to the aforementioned techniques, an advantage of WNJ is that it can measure the P_ET_CO_2_ of the patients, which may help detect problems such as respiratory insufficiency or mechanical failure during anaesthesia in time for timely intervention. A device named the Hague Airway can also monitor P_ET_CO_2_, but its inability to prevent airway obstruction make it limited clinical applications [27]. Furthermore, a high-flow nasal cannula (HFNC), a new oxygen delivery device, is adapted to maintain oxygenation and humidification ventilation. The high flow of gas delivered by the HFNC through the nasopharynx and airways generates a positive end-expiratory pressure, which increases the effective alveolar ventilation, thereby increasing respiratory efficiency and improving oxygenation, but the HFNC is unable to correct the upper airway obstruction induced in the semi-prone position. In addition, HFNC does not directly monitor P_ET_CO_2_, and the apparent SpO_2_ may mask the risk of carbon dioxide accumulation. The LMA® Gastro™ Airway is an airway technique, which could improve airway control, prevent hypoxia and avoid the need for intubation [18]. However, based on our clinical experience, there were difficulties to keep LMA in appropriate position when turned patients to semiprone position. With the advantages of a more open ventilating system, fewer complications, and a lower requirement for spontaneous breathing [3], SJOV has shown its versatility in the emergency airway to support sufficient ventilation [21] and in difficult airways to achieve desirable oxygenation. In our study, the incidence of SRAEs for patients undergoing ERCP under deep sedation were successfully decreased through SJOV via WNJ. Furthermore, lower incidences of muscle twitch, cough and body movement were observed intraoperatively in our study, which indicated that SJOV has the potential to reduce body movements for safer procedures.

Some limitations exist in our study. First, our study is a single-blinded trial with potential biases of outcome assessment, but the objective parameter of hypoxemia might correct for the single-blindness in the present study. Second, the sedation strategy in our study consisted of propofol, remifentanil and dexmedetomidine, so our results cannot be applied to patients with other sedative strategy. Third, we only enrolled patients aged 18 to 65 years with ASA classifications from I to III. The available evidence has demonstrated that the pharmacokinetics and pharmacodynamics of medicine are significantly affected by age [28]. A previous study showed that older age, higher BMI, higher ASA class and longer procedure duration led to higher rates of SRAEs [29]. Therefore, future studies should focus on specific and high-risk patients under deep sedation who may benefit from SJOV in different procedures. Furthermore, the combination use of some useful current monitors such as Oxygen Reserve Index may make the use of SJOV via WNJ safer, which needs further validation.

## Conclusions

In comparison to the nasopharyngeal airway, SJOV via WNJ significantly reduces the incidence of subclinical respiratory depression and SRAEs, especially the incidence of hypoxemia and severe hypoxemia, which is effective to improve oxygenation for patients undergoing deep sedation in the semiprone position during ERCP.

### Electronic supplementary material

Below is the link to the electronic supplementary material.

Supplementary Material 1: Data on anesthesia dosage, anesthesia time, and patient vital signs during the perioperative period

## Data Availability

All data generated or analysed during this study are included in this published article and its supplementary information files.
