# Supplementary material for: Supraglottic jet oxygenation and ventilation improves oxygenation during endoscopic retrograde cholangiopancreatography: a randomized controlled clinical trial
Source: BMC Anesthesiol. 2024 Jan 12;24:21. doi: 10.1186/s12871-024-02406-y (PMC10785419; doi:10.1186/s12871-024-02406-y)
Supplement: Supplementary file 1 — Supplementary Material 1: Data on anesthesia dosage, anesthesia time, and patient vital signs during the perioperative period [file 12871_2024_2406_MOESM1_ESM.docx]

**Supplementary table1: Anaesthesia Characteristics value**

|  | Group  N(Ⅰ)  (n=56) | Group  W(Ⅱ)  (n=56) | Group  WS(Ⅲ)  (n=55) | *P*-value  (Kruskal-Wallis) | Adj.*P*  (Ⅰ vs Ⅱ) | Adj.*P*  (Ⅱ vs Ⅲ) | Adj.*P*  (Ⅰ vs Ⅲ) |
| --- | --- | --- | --- | --- | --- | --- | --- |
| Sedation time  [min;mean(SD)] | 44.50(4.33) | 44.54(4.17) | 44.31(4.57) | 0.924 | - | - | - |
| Procedure time  [min;mean(SD)] | 34.13(4.03) | 33.39(4.07) | 32.27(3.73) | 0.045* | 0.216 | 0.206 | 0.013^#^ |
| Recovery time  [min;mean(SD)] | 7.45(1.67) | 7.61(1.67) | 7.57(1.68) | 0.758 | - | - | - |
| Propofol dose [mg;mean(SD)] | 241.80(40.92) | 236.30(38.43) | 243.70(30.16) | 0.369 | - | - | - |
| Dexmedetomidine dose  [μg;mean(SD)] | 61.21(9.48) | 59.81(8.89) | 61.93(6.85) | 0.306 | - | - | - |
| Remifentanil dose  [μg;mean(SD)] | 145.53(26.83) | 142.23(25.22) | 146.38(20.79) | 0.462 | - | - | - |
| WNJ Placement depth [cm;mean(SD)] | - | 12.20(2.42) | 12.24(2.08) | 0.380 | - | - | - |

Sedation time, time from induction with propofol to opening eyes in response to sound; Procedure time, time from endoscope insertion to withdrawal; Recovery time, time from drug withdrawal until the patient's Aldrete score=8. Adj.*P*, Bonferroni adjust *P* value. The *P*-value was adjusted to 0.05/3≈0.017.

**P*<0.05.

^#^ *P*<0.017.

**Supplementary table2:Intraoperative monitoring data**

|  | Group N  (n=56) | Group W  (n=56) | Group WS  (n=55) | *P*-value |
| --- | --- | --- | --- | --- |
| SBP[mmHg;mean(SD)] |  |  |  |  |
| T1 | 130.68(14.28) | 132.86(17.34) | 130.78(15.25) | 0.742 |
| T2 | 129.32(10.39) | 129.29(15.00) | 126.85(11.33) | 0.370 |
| T3 | 111.05(10.70) | 112.79(12.17) | 113.87(12.21) | 0.464 |
| T4 | 103.30(9.52) | 107.66(12.12) | 106.84(10.98) | 0.141 |
| T5 | 104.45(7.74) | 108.77(11.55) | 107.40(9.95) | 0.103 |
| T6 | 111.41(12.45) | 112.43(12.59) | 110.78(9.63) | 0.866 |
| T7 | 118.34(11.97) | 117.20(13.69) | 114.82(9.90) | 0.348 |
| DBP[mmHg;mean(SD)] |  |  |  |  |
| T1 | 73.79(6.42) | 73.50(10.03) | 70.15(9.25) | 0.026* |
| T2 | 72.02(8.50) | 72.13(9.90) | 69.40(8.32) | 0.259 |
| T3 | 63.00(8.49) | 63.63(10.04) | 64.55(8.55) | 0.557 |
| T4 | 57.98(8.18) | 59.93(11.84) | 61.31(9.83) | 0.304 |
| T5 | 58.57(7.88) | 61.32(10.21) | 63.31(9.60) | 0.077 |
| T6 | 62.57(9.76) | 63.46(9.25) | 63.45(8.87) | 0.867 |
| T7 | 67.29(7.07) | 65.68(8.81) | 65.78(10.77) | 0.264 |
| MAP[mmHg;mean(SD)] |  |  |  |  |
| T1 | 92.75(7.16) | 93.29(10.90) | 90.36(10.04) | 0.058 |
| T2 | 91.12(7.32) | 91.18(10.14) | 88.55(7.86) | 0.180 |
| T3 | 79.02(7.84) | 80.01(9.42) | 80.99(8.53) | 0.573 |
| T4 | 73.09(7.88) | 75.84(11.24) | 76.48(8.63) | 0.170 |
| T5 | 73.86(7.18) | 77.14(9.85) | 78.01(7.86) | 0.052 |
| T6 | 78.85(9.91) | 79.79(9.58) | 79.23(7.78) | 0.852 |
| T7 | 84.30(7.95) | 82.85(9.75) | 81.33(10.20) | 0.162 |
| HR[bpm;mean(SD)] |  |  |  |  |
| T1 | 72.07(9.11) | 71.68(8.01) | 71.85(7.70) | 0.903 |
| T2 | 71.41(8.98) | 70.13(9.47) | 70.13(8.44) | 0.660 |
| T3 | 69.45(9.06) | 68.48(8.01) | 68.87(9.03) | 0.929 |
| T4 | 67.66(9.33) | 68.79(8.30) | 67.62(9.17) | 0.710 |
| T5 | 67.39(8.17) | 67.57(7.99) | 66.82(8.97) | 0.974 |
| T6 | 69.11(8.95) | 69.32(8.48) | 68.24(8.23) | 0.908 |
| T7 | 69.86(8.54) | 70.73(8.99) | 70.49(10.56) | 0.888 |
| BIS[mean(SD)] |  |  |  |  |
| T1 | 89.95(3.32) | 89.61(3.10) | 89.58(3.44) | 0.793 |
| T2 | 79.79(6.34) | 79.48(5.48) | 79.98(6.46) | 0.930 |
| T3 | 51.82(5.81) | 52.84(5.52) | 51.20(6.03) | 0.332 |
| T4 | 52.98(4.91) | 54.21(4.37) | 52.51(4.55) | 0.131 |
| T5 | 53.30(4.83) | 54.11(4.88) | 54.84(5.09) | 0.224 |
| T6 | 53.41(5.98) | 54.77(5.01) | 54.89(5.67) | 0.249 |
| T7 | 77.68(5.37) | 76.39(5.08) | 76.44(5.24) | 0.486 |

Data are presented as the mean (SD).

T1, enter the endoscopy suite; T2, induction with propofol; T3, at the beginning of procedure; T4, 10 min after the beginning of procedure; T5, 20 min after the beginning of procedure; T6, at the end of procedure; T7, at the time of awakening.

Abbreviations: SBP, noninvasive systolic arterial pressure; DBP, diastolic arterial pressure; MAP, mean arterial pressure; HR, heart rate; BIS, bispectral index.

**P*<0.05.
